# Advancing Regenerative Cellular Therapies in Non-Scarring Alopecia

**DOI:** 10.3390/pharmaceutics14030612

**Published:** 2022-03-10

**Authors:** Talagavadi Channaiah Anudeep, Madhan Jeyaraman, Sathish Muthu, Ramya Lakshmi Rajendran, Prakash Gangadaran, Prabhu Chandra Mishra, Shilpa Sharma, Saurabh Kumar Jha, Byeong-Cheol Ahn

**Affiliations:** 1 Department of Plastic Surgery, Topiwala National Medical College and BYL Nair Ch. Hospital, Mumbai 400008, India; dranudeep@gmail.com; 2 Department of Biotechnology, School of Engineering and Technology, Sharda University, Greater Noida 201310, India; madhanjeyaraman@gmail.com (M.J.); drsathishmuthu@gmail.com (S.M.); saurabh.jha@sharda.ac.in (S.K.J.); 3 À La Mode Esthétique Studio, Mysuru 570011, India; 4 International Association of Stem Cell and Regenerative Medicine (IASRM), New Delhi 110092, India; info@iasrmglobal.org (P.C.M.); drshilpas@gmail.com (S.S.); 5 Department of Orthopaedics, Faculty of Medicine—Sri Lalithambigai Medical College and Hospital, Dr MGR Educational and Research Institute, Chennai 600095, India; 6 Department of Orthopaedics, Government Medical College and Hospital, Dindigul 624304, India; 7 Department of Nuclear Medicine, School of Medicine, Kyungpook National University, Kyungpook National University Hospital, Daegu 41944, Korea; ramyag@knu.ac.kr; 8 BK21 FOUR KNU Convergence Educational Program of Biomedical Sciences for Creative Future Talents, Department of Biomedical Sciences, School of Medicine, Kyungpook National University, Daegu 41944, Korea; 9 Department of Paediatric Surgery, All India Institute of Medical Sciences, New Delhi 110029, India

**Keywords:** alopecia, mesenchymal stem cells, regenerative therapy, cellular therapy

## Abstract

Alopecia or baldness is a common diagnosis in clinical practice. Alopecia can be scarring or non-scarring, diffuse or patchy. The most prevalent type of alopecia is non-scarring alopecia, with the majority of cases being androgenetic alopecia (AGA) or alopecia areata (AA). AGA is traditionally treated with minoxidil and finasteride, while AA is treated with immune modulators; however, both treatments have significant downsides. These drawbacks compel us to explore regenerative therapies that are relatively devoid of adverse effects. A thorough literature review was conducted to explore the existing proven and experimental regenerative treatment modalities in non-scarring alopecia. Multiple treatment options compelled us to classify them into growth factor-rich and stem cell-rich. The growth factor-rich group included platelet-rich plasma, stem cell-conditioned medium, exosomes and placental extract whereas adult stem cells (adipose-derived stem cell-nano fat and stromal vascular fraction; bone marrow stem cell and hair follicle stem cells) and perinatal stem cells (umbilical cord blood-derived mesenchymal stem cells (hUCB-MSCs), Wharton jelly-derived MSCs (WJ-MSCs), amniotic fluid-derived MSCs (AF-MSCs), and placental MSCs) were grouped into the stem cell-rich group. Because of its regenerative and proliferative capabilities, MSC lies at the heart of regenerative cellular treatment for hair restoration. A literature review revealed that both adult and perinatal MSCs are successful as a mesotherapy for hair regrowth. However, there is a lack of standardization in terms of preparation, dose, and route of administration. To better understand the source and mode of action of regenerative cellular therapies in hair restoration, we have proposed the “À La Mode Classification”. In addition, available evidence-based cellular treatments for hair regrowth have been thoroughly described.

## 1. Introduction

Alopecia or baldness is a common diagnosis in clinical practice. A variety of causes, including genetics, hormones, autoimmune, trauma, stress, and iatrogenic factors, all play an important part in the pathophysiology of alopecia. Alopecia can be scarring or non-scarring, diffuse or patchy. The most prevalent type of alopecia is non-scarring alopecia, with the majority of cases being androgenetic alopecia (AGA) or alopecia areata (AA). Androgenetic alopecia is a disorder of exaggerated response of hair follicles of the scalp to systemic androgens leading to accelerated patterned hair loss [1,2]. AGA is a progressive condition with a hereditary propensity that is the most prevalent cause of baldness in both men and women. It is characterized by hair follicle miniaturization and inflammation [3,4,5,6,7]. Even though AGA is typically seen in patients in their 20’s and 30’s, disease process begins with the onset of puberty and it progresses thereafter [3,8]. AGA warrants immediate attention from a qualified trichologist because of its psychosocial impact on patients, which in turn may lead to emotional distress and decreased quality of life [9,10,11]. Hair loss pattern in AGA varies between men and women. Male androgenetic alopecia is characterized by bitemporal recession, frontal balding, and balding of the vertex of the scalp, as opposed to female androgenetic alopecia, which is characterized by diffuse thinning of hair and sparing of the frontal hairline [12,13,14].

## 2. Hair Growth Cycle and Its Regulators

Hair is an appendage of the skin. The skin-hair unit has been structurally divided into three subunits, namely interfollicular epidermis (IFE), the hair follicle (HF), and the sebaceous gland (SG). Just beneath the sebaceous gland, near the outer root sheath, we can spot ‘the bulge’, which is a storehouse of stem cells. These stem cells have the innate property of asymmetric self-renewal to produce transit amplifying matrix cells, which encases the mesenchymal cells. These transits amplifying matrix cells move towards the surface to differentiate into epidermal keratinocytes and they move downwards towards the hair follicle matrix to multiply and differentiate into the shaft of hair [15,16]. The adult HF is always a part of a constant hair cycle which consists of three stages, namely (a) catagen—phase of degeneration, (b) telogen—resting phase and (c) anagen—growth phase.

The cradle of proliferation during the anagen phase is HF stem cells (HFSCs). The matrix cells undergo apoptosis during the catagen phase which moves the dermal papilla (DP) towards the epidermis below the hair germ, it is the early descendants of bulge stem cells. DP maintains HFSCs in a dormant state and proficient for the next hair cycle [17].

**Figure 1:**
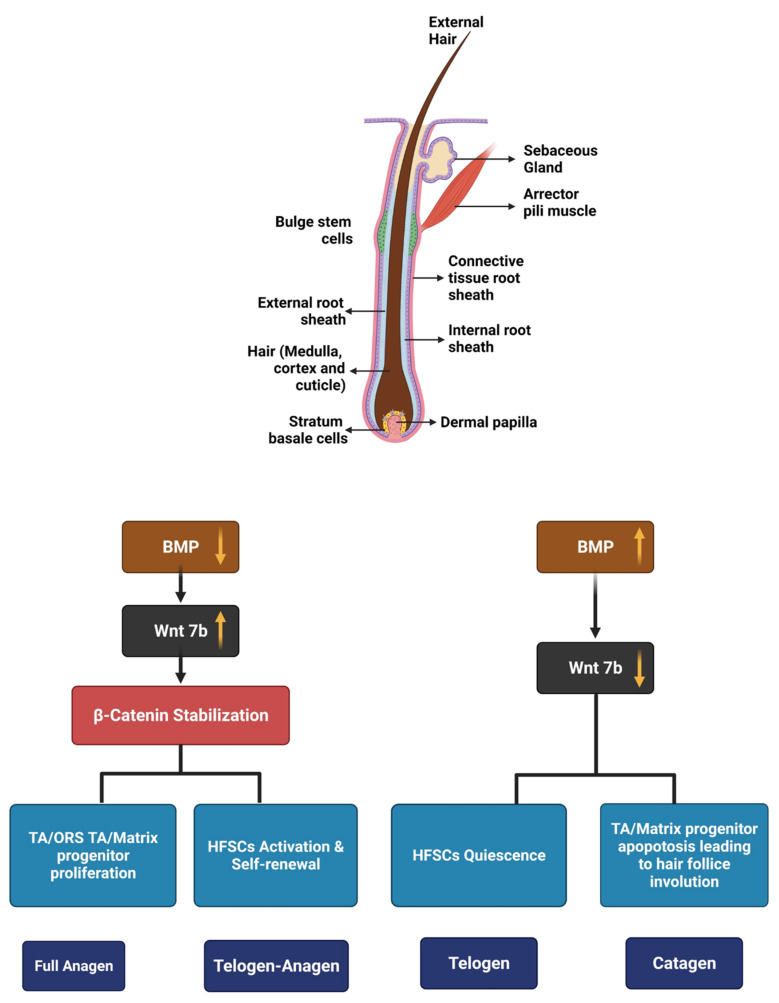
Structure of hair follicle and the molecular mechanisms involved in hair cycle. (BMP—bone morphogenetic protein, TA—transit amplifying, ORS—outer root sheath, HFSCs—hair follicular stem cells). Created with BioRender.com (accessed on 27 January 2022).

SG and IFE are not maintained by HFSC, but they will help in the regeneration of the epidermis and SG after wounding. Based on their location with respect to the basal lamina, HFSCs are divided into basal and suprabasal HFSCs [18]. The interaction between epithelial and mesenchymal components is important to regulate HFSCs. Wnt signaling helps in the proliferation by stabilizing β-catenin, which translocate to the nucleus and complexes with LEF1 to form a transcriptional activating complex leading to proliferation. Hair follicles are maintained in the dormant state by Lef-1/Tcf-3 [19]. In the adult HF, the accumulation of β-catenin in the nucleus of HFSCs is associated with telogen to anagen transition, this signifies the importance of Wnt signaling in the self-renewal capacity of the stem cells [20]. Wnt/β-catenin signaling and LEF1 in the bulge are very important for matrix cell differentiation towards the shaft [21]. Despite this, the source of the Wnt ligand is not easy to elucidate [22]. The BMP pathway inhibits HF morphogenesis and adult HFSC proliferation (Figure 1) [23,24]. BMP ligands and the antagonist noggin are balanced by mesenchyme [19]. Heightened cycling of HFSCs is seen when the BMPR1a receptor is inactivated in the HF and it impairs differentiation. The Hedgehog and Notch signaling pathways are also implicated in the proliferation and differentiation of HF [25].

## 3. Treatment of Non-Scarring Alopecia and Adverse Effects

Conventionally, AGA treatments aim at lowering the levels of dihydroxy testosterone by using 5 alpha-reductase inhibitors, minoxidil and finasteride, a potassium channel opener [26]. Minoxidil (topical 5% solution/foam) and finasteride (oral 1 mg/day) are the first-line therapies for AGA.

Finasteride is an inhibitor of 5 alpha-reductase type 2, which decreases the concentration of DHT in serum and scalp and, hence, increases hair growth. It has to be taken for at least 1 year to notice hair growth and has to be continued to maintain the hair growth. The effect of regrowth will be lost if the drug is stopped for 6–9 months. Erectile and ejaculatory dysfunction are among the many negative effects of finasteride. Gynecomastia, testicular pain, and depression are the rare adverse effects of finasteride [27,28]. Finasteride may interfere with the levels of prostatic specific antigen (PSA), it is shown that PSA level decreases considerably in patients taking finasteride [29].

Minoxidil is a potassium channel opener. It is a vasodilator and induces vascular endothelial growth factor (VEGF) to increase vascularity and dermal papilla size [30,31]. The response to treatment is variable. It requires 12–18 months to assess the efficacy of minoxidil. It has to be continued life long and the effect ceases with the stoppage of treatment. There might be exaggerated hair fall in the initial 2 months (telogen to anagen transition phase), but it improves over 2 months. Adverse effects include contact dermatitis, irritant dermatitis, and hypertrichosis over the face [32,33]. Other new modalities include platelet-rich plasma (PRP), mesotherapy, lasers, micro-needling of the scalp, and Janus kinase (JAK) inhibitors. When patients are keen on immediate results, they resort to hair transplantation, i.e., follicular unit transplantation (FUT) or follicular unit extraction (FUE).

It is clear from the studies that first-line therapies for AGA that:

1. They have to be continued lifelong, which decreases the compliance;
2. The regrowth ceases with the discontinuation of therapy;
3. They are associated with several adverse effects leading to temporary morbidity.

Alopecia areata (AA) is a non-scarring chronic, immune-inflammatory disorder of hair follicles. The most typical manifestation of AA is the presence of localized patches of hair loss on the scalp, but severe cases can result in generalized hair loss throughout the body [34]. AA affects about 2% of the general population at some point in life [35]. The disruption of the hair follicle’s immune privilege is regarded to be a key element in the pathophysiology of AA. It is centered on a lymphocytic infiltration in and around the hair follicle’s bulb or lower part. Despite various therapeutic options, such as corticosteroids (topical, intralesional, oral), tacrolimus, minoxidil, contact immunotherapies like squaric acid dibutyl ester, diphencyprone, and photo(chemo)therapy using UVA and psoralens, there is no cure for AA [36].

It is obvious that the therapies for AA:

1. Have unpredictable outcome;
2. Have no permanent cure;
3. Are associated with significant adverse reactions.

This has led researchers to explore regenerative therapies for hair restoration in non-scarring alopecia. Being autologous/allogenic, they are free from adverse effects with good patient compliance. The regenerative modalities explored are PRP, amniotic fluid, adipose-derived stem cells, follicular micrograft, bone marrow cells, cord blood and Wharton jelly.

Regenerative therapies in non-scarring alopecia can include cells, which can produce factors inducing hair growth or the products of the cells which can be isolated and used. Cells as such are difficult to maintain in culture for transplantation whereas growth-factors secreted by the cells in the medium are easy to transport and is less expensive compared to cellular therapy as such. Hence, it is essential to classify the regenerative therapies into growth factor-rich and stem-cell rich, which will aid in better understanding, clinical utility and for further research. Therefore, we suggest this “A La Mode Classification” (Table 1).

**Figure 2:**
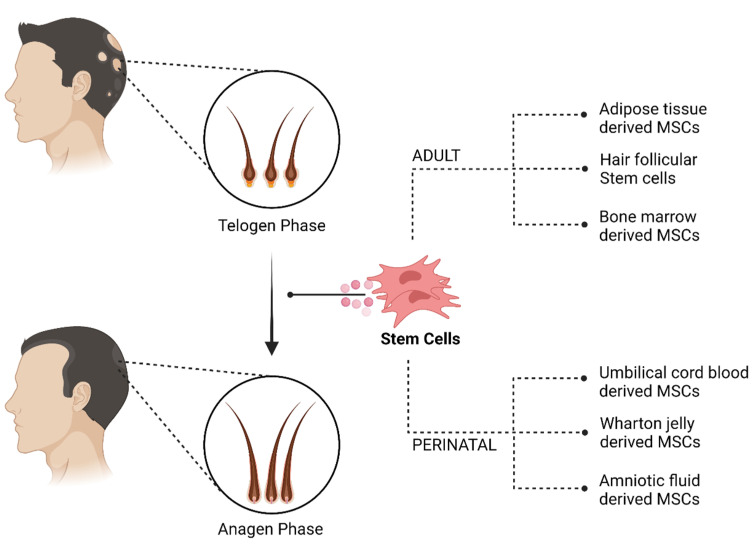
Cellular therapy for non-scarring alopecia. Created with BioRender.com (accessed on 1 March 2022).

In this article, we will review the cellular therapies used to treat non-scarring alopecia, as shown in Figure 2, their mechanism of action and the recent studies to validate its efficacy. In addition, studies on conditioned medium are briefly mentioned at places to reiterate the potential of cellular therapy.

## 4. Cellular Therapy

### 4.1. Adult Stem Cells

MSCs are the most dynamic, immature, diverse, and multipotent stromal progenitor cells. They have a morphology similar to fibroblasts and have the capacity for trans-differentiation into a range of tissues of ectoderm, endoderm, and mesodermal origin. MSC clonal heterogeneity is demonstrated in terms of variable differentiation, regeneration and proliferative capability in both in vivo and in vitro studies. MSCs express non-differentiating cell surface markers, such as CD146 or CD200. MSCs are found in bone marrow, the placenta, the umbilical cord, fat, menstrual blood, molar teeth and amniotic fluid [61]. As there are multiple sources of MSCs, it is of immense importance to define an MSC. In 2006, Mesenchymal and Tissue Stem Cell Committee of the International Society for Cellular Therapy has proposed three minimum criteria to define an MSC. They are as follows:

1. MSC must be plastic adherent when maintained under standard culture conditions.
2. Expression of CD105, CD73 and CD90, and lack expression of CD45, CD34, CD14, or CD11b, CD79a, or CD19 and HLA-DR surface molecules.
3. They must differentiate to osteoblasts, adipocytes and chondroblasts in vitro [62].

MSCs are more adaptable in nature, allowing them to switch from one differentiation path to another as influenced by growth factors, cytokines, and chemokines. The conversion of chondrocytes into osteoblasts and osteoblasts into chondrocytes has been demonstrated in the literature (trans differentiation) [63]. Pathway conversion from one cell lineage and another cell lineage can explain the concept of de-differentiation. De-differentiation lineage conversion is based on a phase space model or a noise-driven stem cell differentiation model. Self-renewal and specialization characteristics are inversely proportional. A cell’s plasticity eliminates the necessity for cells to have a constant capability for self-renewal. Plasticity necessitates a cell’s whole differentiation potential in order to form the final product. Lineage priming is another feature of MSCs. The pathway is primed to follow a certain lineage to differentiate into a final cell of interest as a result of the action of growth factors and transcription factors.

The application of stem cells at the site of action entails advocating in two methodical approaches are either direct delivery to target site or systemic administration. Direct delivery implies that harvested stem cells from any source either bone marrow or adipose tissue or placenta or umbilical cord can be injected or implanted at the exact site for regeneration. The procedure of direct delivery eliminates the delay of stem cells to reach target site of action and hastens the regenerating and rejuvenating process. In the treatment of alopecia, direct delivery has been useful and studied with good results. The other mode of stem cell delivery is systemic delivery (intramuscular, intravenous, intra-articular), where stem cells will be harvested and isolated from its source and will be cultured in laboratory media to exponentially escalate stem cell count to be transplanted [64]. Regeneration of hair follicles caused by MSCs may be due to reversal of the pathophysiology of alopecia, regeneration of partially destroyed hair follicles or stem cell induced formation of new hair follicles [65,66,67].

#### 4.1.1. Adipose Tissue-Derived Cells

MSCs were traditionally obtained from bone marrow, but with advancements in research, adipose derived stem cells (ADSCs), located in fat tissues including subcutaneous fat tissue, are now easily accessible compared to other sources of MSCs [68,69]. Subcutaneous tissue mainly comprises of adipocytes along with other cells like MSCs, fibroblasts, and endothelial cells. The adipose tissue is a warehouse of regenerative molecules, various regenerative products that can be derived out of adipose tissue are nano fat, stromal vascular fraction (SVF), MSCs, adipose derived stem cells-conditioned medium (ADSC-CM), and extracellular vesicles (EV).

Nano fat refers to adipose graft with size less than 400 to 600 microns. They are obtained by mechanical fragmentation and filtration. They are rich in ADSCs with few adipocytes and associated cells whereas SVF mainly comprises of MSCs, endothelial cells, pericytes, immune cells and stromal cells but devoid of any adipocytes. MSCs can also be isolated from the adipose tissue and cultured in pure form. SVF and MSCs are differentiated by their respective specific surface markers. ADSC-CM are rich in growth factors and the commercially available preparation is known as AAPE (advanced adipose derived stem cell protein extract) which is obtained from culturing ADSCs and then the proteins secreted by them are extracted in their lyophilized form [39,70].

The stromal vascular fraction is made up of a mix of stem cells from adipose tissue, endothelial precursor cells, mature endothelial cells, lymphocytes, pericytes and pre-adipocytes [71,72]. SVF contains roughly 0.01% stem cells. SVF’s diverse biological components boost the permeability of endothelial and epithelial progenitor cells, enhancing the regeneration capacity of diseased and damaged tissues. Among other stem cell isolates, SVF is said to have the highest therapeutic efficacy. Traktuev et al. found that VEGF in SVF aids migration and survival of endothelial progenitor cells [73,74].

SVF has the appearance of fibroblasts and the characteristics of MSCs. SVF promotes differentiation of diverse cell lineages due to its MSC-like properties. SVF’s cellular composition contains both HSCs (CD-34 and 45) and MSCs (CD-105 and 146) surface markers. SVF cells express several of the same cell surface markers as bone marrow-derived MSCs, including CD-24, 29, 31, 44, 45, 71, 90, 105/SH2 and SH3. SVF has anti-inflammatory and antiandrogenic properties. In addition, being rich in MSCs, they help in hair restoration [51]. The key function of MSCs is to maintain homeostasis by facilitating recovery after any insult or injury [75]. ADSCs have the ability to differentiate into tissues of mesenchymal origin. They are also known to secrete bioactive molecules, such as VEGF, hepatocyte growth factor (HGF), insulin-like growth factor (IGF), and platelet-derived growth factors (PDGF). These growth factors act on the surrounding cells to mediate their functions. They have an important role in neovascularization, which is crucial in the pathophysiology of many types of alopecia. The significance of adipose tissue in alopecia came in to lime light because of its ability to increase vascularity of the scalp when autologous fat was injected to the scarred scalp before hair transplantation. When scalp was pre-treated with adipose tissue injections, it bled more during hair transplantation. The same principle of increased vascularity was extrapolated to treat alopecia [51,76,77]. Many studies have shown considerable results in the treatment of alopecia with adipose derived cells and products [78,79].

AD-MSCs express CD34 for roughly 8–12 cellular doublings in culture [71]. Numerous hypotheses exist about the role of pericytes in the stem cell characteristics of SVF. Pericytes are seen in both MSCs and ADSCs, according to Szoke et al. [80]. However, Traktuev et al. and Crisen et al. claim CD34+ and CD34− pericytes to be the identities of ADSCs, respectively [74,81].

When 1 mL/cm^3^ of SVF was injected subcutaneous to scalp, there was significant increase in hair density (31 hair/cm^2^). Combination of SVF with fat graft increased the density to 44.1 hair/cm^3^ compared to the baseline [82]. Similarly, intradermal injection of 5 mL SVF in 20 patients showed statistically significant increase in hair density and diameter with improvement in hair pull test at 6 months [83]. SVF-enriched autologous fat demonstrated 14% increased hair count at 6 months [84]. ADSC-CM has also shown statistically significant increase in hair density and thickness when 4 mL was used via microneedle roller weekly for 12 weeks [85] [86]. Similar results were seen when monthly intradermal ADSC-CM was administered for 6 months [87]. In a study conducted in Korea, a single injection of autologous SVF to scalp led to statistically significant increase in hair density at 3 and 6 months; improvement in keratin score and hair thickness was also noted [88]. Fakuoka et al., also documented reduction in hair thinning, increased hair quality and number of hair with ADSC-CM [39]. Addition of PRP to SVF to get PRS (platelet rich stroma) has also been studied in 10 patients with AGA. A single dose of PRS significantly improved hair density. Interestingly, this study also demonstrated new hair growth in hyperkeratotic plugged non-functioning hair follicles [89]. It is challenging to isolate and quantify the biological components of SVF, despite its greater translational potential, in regenerative medicine. Several researches have suggested that SVF has a high potential for regenerating tissues [71].

#### 4.1.2. Hair Follicular Stem Cells

Adult stem cells have the innate ability to regenerate damaged or senescent cells which is mediated through intrinsic mechanisms which in turn will control the expression of genes via transcription. Stem cells achieve homeostasis by responding to their surrounding and also ambience based self-signaling. These stem cell-microenvironment interactions moderate cell growth, differentiation and also the renewal and maintenance of stem cell pool till death of tissue [90,91,92,93].

HFSCs found in the bulge region of hair follicle include epithelial and melanocytic stem cells. They are mostly dormant but they have the innate ability to migrate, proliferate and differentiate in order to maintain homeostasis [94] The latent HFSCs of the bulge region spawn ‘primed stem cells’ (also known as early progenitor cells) which later produce progenitor cells in hair matrix, these progenitor cells are fast multiplying. The progenitor cells further differentiate and move towards the surface to form inner root sheath and shaft [95] With the ongoing differentiation of matrix cells, the stem cells located in the bulge undergo self-renewal in the anagen phase and they will remain in the bulge region to become dormant again [96,97] Some dormant HFSCs move out of the bulge to form a new bulge and a new set of primed stem cells to start a fresh cycle. This recurrent activity occurs during anagen, catagen and telogen [98] Not just hair follicle homeostasis, HFSCs also play a role in the generation of interfollicular epidermis, SG, and aid in wound healing [99,100,101].

In AGA, it is shown that HFSCs are normal in number but there is decreased pool of actively multiplying progenitor cells. This observation clearly conveys that the pathology is not in the number of HFSCs but with the regulator of these stem cells by activating or inhibiting it [102].

A placebo-controlled study was conducted on 11 patients with AGA Norwood stage 3–5 to quantify the isolated HFSCs microscopically and to know the effect of HFSCs procured by centrifugation of fragmented scalp hair follicle obtained through punch biopsy without any culture. 3728 ± 664.5 cells were present in each scalp suspension. CD44 + MSCs derived from hair follicles amounted for 5% ± 0.7%, while CD200+ hair follicle epithelial stem cells from the bulge accounted for 2.6% ± 0.3%. Mean hair count and hair density (29% ± 5% vs. placebo 1% increase) improvement was observed 23 weeks after the last treatment [67]. Likewise, another placebo-controlled study was conducted to know the efficacy of autologous PRP (A-PRP) and HF-MSCs obtained by using Rigeneracon device in AGA. Patients treated with A-PRP showed increased mean hair count and hair density (31% ± 2%) 12 weeks after the last injection. HF-MSCs group showed significant increase in mean hair density of 30% ± 5% (after 12 weeks), 29% ± 5% (after 23 weeks) when compared to placebo (<1%) [103]. Another double blinded study with HF-MSCs reiterated the findings of previous studies which showed increased hair density and count. Interestingly, dynamic hair loss was noted in 6 patients after 26 months [99]. Increased hair density was seen when micro grafts produced from scalp tissue including HD-AFSCs were employed [52].

#### 4.1.3. Bone Marrow-Derived Cells

Bone marrow is the conventional source of MSCs. Many animal models have shown progression from telogen to anagen phase after intra dermal injection of bone marrow derived mesenchymal cells (BM-MSCs) and they also induced genes involved in hair regrowth [104]. Both BM-MSCs and ADSCs are rich in MSCs but their concentration and differentiation abilities are different. BM-MSCs have more of osteogenic potential and ADSCs being primarily angiogenic. BM-MSCs retrieval is relatively invasive and hence it is underutilised for hair restoration [105]. BM-MSCs and follicular stem cells (FSC) are known for their role in the treatment of alopecia. Occipital hair follicles are innately resistant to AGA and hence are preferred when FSCs are utilized [56]. The preparation of bone marrow aspirate concentrate (BMAC) follows double centrifugation technique called differential or density centrifugation [56,106].

In the normal morphogenesis of skin and its appendages, Wnt and BMP signaling plays a vital role. After culture MSCs detach and form dermal papilla like tissue (DPLT). The DPLT was similar to the human dermal papilla cells (DPC). Growth factors, anti-inflammatory and angiogenic factors also play a role in improving hair growth. The progenitor cells account for 0.001% to 0.01% of BMAC by gradient centrifugation. Growth factors, such as PDGF, TGF-β and BMP-2 and -7 have been shown to have anabolic and anti-inflammatory effects, and it is worth noting that these are present in higher concentrations in BMAC. MSCs induce the synthesis of IL-1Ra and IL-1 molecules in substantial quantities, and these molecules carry out the bioactivity of blocking IL-1 catabolism [107,108,109].

Yoo et al. investigated the use of MSCs from bone marrow and umbilical cord in human hair proliferation in vitro. DPLTs produced by their method had characteristics similar to actual DPC (Size, shape and protein structure), which was demonstrated by microscopy and immunohistochemistry. Transplanted DPLTs also stimulated the growth of new HFs in athymic mice [109].

There is only one human study published in relation to the utilization of BMAC with good sample size. The purpose of this study was to assess the efficacy of autologous bone marrow mononuclear cells (BMMC) and FSC in AA and AGA. It was carried out on 40 patients, who were divided into four groups of ten each namely patients with AA receiving intradermal BMMC, patients with AA receiving intradermal FSC, patients with androgenetic alopecia receiving intradermal BMMC and patients with AGA receiving intradermal FSC. There was significant improvement 6 months after stem cell therapy, which was evaluated using immunostaining and digital dermoscopy. There was no difference between BMMC and FSC groups and no adverse effects were reported [56].

There are very few human studies to evaluate the efficacy of BMAC and BMMC, the biology of BMAC has to be well understood for clinical applications. Future of hair restoration lies in the regenerative molecules. Bleeding, infection and persistent pain are the known adverse effects during bone marrow aspiration. BMAC being autologous and easily accessible will play a major role in future.

### 4.2. Perinatal MSCs

#### 4.2.1. Umbilical Cord Blood-Derived Cell

One of the well-known sources of MSCs is umbilical cord. MSCs derived from human umbilical cord blood (hUCB-MSCs) have the capacity to repair tissue [110,111] They can directly replace the damaged and senescent cell or act through their secretions, paracrine way. This paracrine action is the main mechanism by which hUCB-MSCs act [112,113,114] Their therapeutic efficacy is based on the action on DPCs. hUCB-MSCs are isolated from umbilical vein [57].

When these cells are cultured in an appropriate conducive medium, they are known to secrete growth factors and cytokines having paracrine action. These paracrine actions will induce angiogenesis and induce hair regrowth. These secreted factors are secretome and extracellular vesicles. They will get concentrated in the culture medium as known as ‘conditioned medium’ (CM). CM is easier to incorporate into clinical practice than MSCs because of decreased production costs and feasible transportation [57,87,115] Therapeutic role of CM is based on its action of growth factors. It enhances VEGF, PDGF, IGF-2 and EGF down-regulation, mediated via Wnt/β-catenin pathway in DPCs [116,117] DPCs enhance hair regeneration by signaling epidermal stem cells in the bulge area. In addition, glucocorticoids inhibit hair growth, this effect was inhibited by hUCB-MSCs [118,119,120,121].

An in vitro and in vivo animal investigation was carried out to investigate the mechanism of action of hUCB-MSCs in hair follicle regeneration. They found out that these stems cells increased regeneration of new follicles and improved onset of anagen phase. They also noted that the proteins involved in increased growth of hair. Insulin like growth factor binding protein 1 (IGFBP-1) and VEGF were upregulated by hDPCs when they were cultured with hUCB-MSCs in vitro. They concluded that paracrine mechanism plays a vital role [57]. Another double blinded placebo-controlled study was conducted to assess the efficacy of CM derived from hUCB-MSCs. They used topical 5% CM obtained from hUCB-MSCs, which was pre-treated with lithium chloride (LiCl) and TGF-β1. It was observed that the hair density, growth rate and thickness increased significantly. In addition, MSCs secreted migration inhibitory factor (MIF), which regulate VEGF secretion from DPCs via β-catenin and p-GSK-3β [SER9] pathways. Effects of the primed-hUCB-MSCs derived CM were paracrine in nature [40].

#### 4.2.2. Wharton Jelly Derived Cell

The umbilical cord’s vessels are encased in Wharton jelly, a type of connective tissue. This connective tissue is a ware house of UC-MSCs, also known as Wharton’s jelly MSCs (WJ-MSCs) and the matrix is rich in growth factors like, IGF, FGF and TGF-β [122,123,124,125,126,127]. Wharton jelly can be extracted from three different parts of the umbilical cord: sub-amniotic, perivascular, and intervascular. Their structure, function, and immunohistochemistry are all different. Their doubling time is less with high proliferation index ex vivo, up to 300 times yield with six to seven passages. Interestingly with no abnormal karyotypes [128,129,130,131,132,133] WJ-MSCs have tumor suppressor genes in abundance and are not associated with any teratomas. They are also known to secrete hematopoietic cytokines [134] WJ-MSCs can be isolated and expanded using a technique illustrated by Can et al. [135] Aljitawi et al. [136] and Wand et al. [137].

According to Jadalannagari et al., WJ-MSCs have the ability to develop into ectodermal lineage cells [138] When WJ-MSCs were expanded on decellularized Wharton’s jelly matrix (DWJM) in the presence of media promoting bone differentiation, they yielded CK19 positive cells with structures resembling hair as demonstrated by Aljitawi et al. [136].

A study was conducted on SCID mice to demonstrate the efficacy of WJ-MSCs on wound healthy. When amniotic membrane scaffold which was decellularized was implanted with WJ-MSCs used in skin injury, wound healing improved with decreased scarring associated with growth of hair. Also, with considerable improvement in biomechanical properties. They also demonstrated successful expansion of WJ-MSCs using human platelet lysate. They conclude that the stemness of fetal MSCs was more than the BM-MSCs and the amount of immunomodulatory secretion in proinflammatory state was more from the fetal MSCs in comparison to the adult MSCs [139]. Similarly, cultured UC-MSCs in appropriate medium-formed structures resembling the normal dermal papilla along with protein expression pathways similar to DPCs. Intradermal infusion of these DPLT along with outer root sheath cells to athymic nude mice demonstrated increased hair growth after 6 weeks [140]. Another study was carried out on athymic mice to elucidate the prospective of MSCs (BM and UC) as an alternative to DPC for treating alopecia. In vitro culturing of MSCs formed DPLT under special medium. Histologic and immunohistochemical analyses showed that DPLT were similar to native human scalp DPCs. Reconstructed DPLTs when transplanted to athymic mice showed the potential to induce hair follicles [109]. In a study to optimize the reconstruction of DPLTs, it was discovered that HGF is required for differentiation, although it is costly. This study also demonstrated that EGF is an inexpensive alternative [141]. 67% growth was observed when intradermal injection of allogenic WJ-MSCs was injected for AA. There were more hair growth dynamics during the first 3 months (average 52.2%) when compared to hair regrowth following 3 months (average 32%). All patients showed improvement without any adverse effect [58].

#### 4.2.3. Amniotic Fluid-Derived Cell

Amnion refers to a sac enclosing a developing embryo, the sac is filled with amniotic fluid (AF). AF composition and amount varies with the gestational age. During early gestation, osmotic gradient maintains the AF and in the later half it comprises of fetal urine and secretions from the respiratory tract [142,143] AF has variety of cells from the embryo (amniotic membrane, genito-urinary, gastrointestinal and respiratory tracts). Cellular component of AF gradually increases with the gestational age [59,144].

Cellular component comprises of amniocytes, epitheloid and fibroblastic cells. Recent studies have isolated amniotic fluid stem cells (AFSC) and amniotic fluid-MSCs (AF-MSCs). They can be cultured from the cellular component [145] AFSCs account for 1% of cells. They display markers associated with embryonic and adult stem cells, including CD117, and are capable of differentiating into any of the three germ lines [146,147] AF aspirated during mid-trimester of pregnancy is a good source of MSCs. Most of the studies have utilized AF-MSCs obtained during the second trimester (16–28 weeks) through amniocentesis [148,149,150,151,152] Very few studies have utilized AF isolated during first trimester and during parturition [153,154] AF-MSCs are isolated from AF by centrifuging and then culturing the cells at specific conditions, then the cells are sorted using CD117. It is difficult to expand AF-MSCs on a large scale because of the loss of differentiation potential [146,155,156].

AF-MSCs secrete MCP-1, IL-8, IL-6, EGF, SDF-1 and VEGF into the conditioned medium to exert its action, which plays a vital role in angiogenesis [157] MCP and IL-6 secreted by AF-MSCs help in immune modulation [158] Even though cells derived from AF are used along with other regenerative molecules in clinical practice for alopecia, not many randomized blinded clinical studies are available to support the use of AF-MSCs in particular.

In a study conducted by Park et al., the effects of Nanog overexpression in AF-MSCs was assessed for DPCs and regrowth of hair in vivo. They used conditioned medium derived from Nanog overexpressing AF-MSCs (AF-N-MSCs). There was upregulation BMP, FGF, IGF, PDGF and WNT families by the presence of AF-N-MSCs, among them bFGF, IGF, Wnt7a, and PDGF-AA are implicated in hair regeneration by activating DP cells. They discovered that overexpression of Nanog in MSCs triggered telogen to anagen transition and increased hair follicle density by upregulating genes, such as ALP, LEF1, and versican [159] (Table 2).

## 5. Conclusions

Alopecia is a difficult condition to treat as hair fall relapses with the discontinuation of conventionally available FDA approved treatments. We have broadly classified the available regenerative therapies (“A La Mode Classification”) as growth factor-rich and stem cell-rich for better understanding and clinical utility. We have summarized the available regenerative cellular therapies, their mechanism of action and the available clinical trials in the treatment of non-scarring alopecia. Almost all the studies utilizing cellular therapies have shown significant improvement in hair regrowth with no adverse effects. MSCs are the core of these cellular therapy because of their angiogenic and immunomodulatory function, which will aid in hair regrowth. These regenerative therapies can be a boon in the treatment of alopecia if utilized properly.

It is very much clear that there are multiple sources of MSCs available for cellular therapy. Apart from the sources described in this article to treat alopecia, there are few other unexplored sources which can be used to isolate MSCs like dental pulp, peripheral blood, synovium and synovial fluid, endometrium, human foreskin, skin biopsy and muscle [160]. Further research is essential to know the potential of these sources in hair regeneration and the adverse reactions associated with them. Each of these MSCs have their own pros and cons with respect to isolation, differentiation capacity, cell count, and the possible adverse reactions. Further studies are required to compare the efficacy of MSCs derived from various sources. CM has the advantage of low cost, easy storage and transport. Hence, CM is seeking significant attention from clinical researchers. Standardization of isolation technique, culture medium used, dosage, route and depth of injection, clear cut indications and contraindications along with universally acceptable documentation of results have to be gracefully addressed with good quality randomized controlled trials.

## Figures and Tables

**Table 1:** “A La Mode Classification” of regenerative therapies [37,38,39,40,41,42,43,44,45,46,47,48,49,50,51,52,53,54,55,56,57,58,59,60].

Stem Cell-Rich	Growth Factor-Rich
**I. Adult stem cells** A. Adipose derived stem cell (ADSC) 1. Nanofat 2. Stromal vascular fraction (SVF) B. Hair follicular stem cell (HFSCs) 1. Autologous micro grafts [Human intra and extra dermal adipose tissue derived hair follicle stem cells (HD-AFSCs)] 2. Cultured HFSCs i. Hair follicle-derived MSCs (HF-MSC) ii. Hair follicle epidermal stem cells (HF-ESC) C. Bone marrow derived 1. Bone marrow mononuclear cells (BMMC) 2. Bone marrow aspirate concentrate (BMAC) **II. Perinatal stem cells** A. Umbilical cord blood derived B. Wharton jelly MSCs (WJ-MSC)/Umbilical cord MSCs (UC-MSC) 1. Sub amniotic. 2. Perivascular. 3. Intervascular. C. Amniotic fluid derived D. Placental MSC	**I. Platelet-rich plasma (PRP)** A. Autologous activated PRP (AA-PRP) B. Autologous non-activated PRP (A-PRP) **II. Autologous growth factor concentrate (GFC)** **III. Conditioned medium (Secretomes)** A. ADSC-CM (AAPE) B. hUCB-MSC-CM C. AF-MSC-CM D. HF-MSC-CM E. BM-MSC-CM (Genetically engineered) **IV. Extracellular vesicles** A. Exosomes i. New-born foreskin stem cell ii. DPC iii. BM-MSC B. Exosome-like (Ginseng) C. Microvesicles **V. Placental extract**

**Table 2:** Summary of studies on cellular therapies in hair regrowth.

Title	Study Type	Sample Size	Active Agent	Results	Remarks
**Adipose tissue derived stem cells (ADSC)**					
Hair follicle growth by stromal vascular fraction enhancedadipose transplantation in baldness [82]	Pilot case series in humans (MPHL—Grade 2 to 6 and FPHL—Grade 1 to 3)	9	Adipose tissue enriched SVF	when fat + SVF was used, mean increase was 31 hair/cm^2^. Whereas, for fat alone, it was 14 hairs/cm^2^.	6 month follow up was available for only 6 patients
Cellular therapy with human autologous adipose-derived adult cells of stromal vascular fraction for alopecia areata [83]	Human clinical study (Alopecia areata: Grade 1 or 2)	20	Adipose derived stromal vascular cells	It was noted that there was statistically significant increase in hair density, hair diameter and pull test value.	-
Stromal Vascular Fraction Enhanced Adipose Transplantation in Hair Loss: Early Experience & Active Phase II FDA Investigation [84]	Prospective, single blinded human clinical trial (AGA; Grade 1 to 4)	9	Adipose tissue enriched SVF	Compared to baseline, there was a 14 percent increase in the number of hairs (*p* = 0.01), with a mean difference of 28 hairs and a 34 percent increase in the anagen percentage (*p* = 0.09).	Only 6 patients were analyzed at 6 months as 3 lost follow up.
Clinical use of conditioned media of adipose tissue-derived stem cells in female pattern hair loss: a retrospective case series study [85]	Retrospective, observational human study (FPHL)	27	ADSC-CM (AAPE)	The density of the hair rose from 105.4 to 122.7 hairs/cm^2^ (*p* = 0.001). The thickness of the hair increased from 57.5 μ to 64.0 μ (*p* = 0.001).	Dose: Once per week for 12 weeks
Hair Regeneration Treatment Using Adipose-Derived Stem Cell Conditioned Medium: Follow-up With trichograms [87]	Prospective human study on alopecia	22 (Half side comparison: 10)	ADSC-CM (AAPE)	- Hair count increased considerably following therapy in both male (including those who did not receive finasteride) and female patients. - The rise in hair count was substantially greater on the treatment side than on the placebo side in the half-side comparative study.	Dose: 6 sessions every 3–5 weeks
Innovative method of alopecia treatment by autologous adipose-derived SVF [88]	Clinical human study(AGA)	9	Autologous SVF	- Statistically significant hair density at 3 and 6 months. - Improvement in keratin score and hair thickness was noted.	A single dose of Autologous SVF was administered
Introducing Platelet-Rich Stroma: Platelet Rich Plasma (PRP) and Stromal Vascular Fraction (SVF) Combined for the Treatment of Androgenetic Alopecia [89]	Clinical human study (AGA)	10	PRP + SVF (Platelet rich stroma)	- There was statistically significant increase in hair density at 6 and 12 weeks. - New hair growth was also observed in hyperkeratotic plugged non-functioning hair follicles.	A single dose of Autologous PRP + SVF was administered
**Hair follicular stem cells (HFSC)**					
Stem cells from human hair follicles: first mechanical isolation for immediate autologous clinical use in androgenetic alopecia and hair loss [67]	Prospective human study (AGA)	11	HFSC obtained by mechanical centrifugation of punch biopsy from hair follicle	- After 23 weeks, there was a 29% ± 5% increase in hair density in the treated area. - Each suspension of scalp tissue contained approximately 3728.5 ± 664.5 cells. HF-MSC (CD44+ from DP): 5% + 0.7% and HF-ESC (CD200+) from the bulge was about 2.6% + 0.3%.	Primary outcomes were microscopic identification and counting of HFSCs.
Autologous Cellular Method Using micrografts of Human Adipose Tissue Derived Follicle Stem Cells in Androgenic Alopecia [52]	Retrospective observational case-series, randomized, evaluator-blinded, placebo controlled, half-head group study in human (AGA)	33	Autologous cell biological technique (A-CBT) based on micro-grafts containing Hair Follicle Mesenchymal Stem Cells (HF-MSCs)	- Hair density improved, with a mean increase of 33% ± 75% at week 23 and 27% ± 35% at week 44. - The increase in the number of hair follicles per mm^2^ after 11 months was statistically significant (*p* = 0.05).	Dose: 3 injections at 45 days interval
Platelet-Rich Plasma and Micrografts Enriched with Autologous Human Follicle Mesenchymal Stem Cells Improve Hair Re-Growth in Androgenetic Alopecia. Biomolecular Pathway Analysis and Clinical Evaluation [103]	Retrospective observational case-series in humans (AGA)	21 (HF-MSC) 57 (A-PRP)	HF-MSC And A-PRP	- 31% ± 2% increase in hair density in A-PRP group. - In HF-MSC group, 30% ± 5.0% increase in hair density after 12 weeks, 29% ± 5.0% in 23 weeks.	- Dose: 2 sessions 2 months apart for HF-MSC, whereas, 3 sessions 30 days apart for PRP - All cases received low-level led treatment (LLLT). 15 days following each treatment and every three weeks thereafter until six months post-treatment
Autologous Micrografts from Scalp Tissue: Trichoscopic and Long-Term Clinical Evaluation in Male and Female Androgenetic Alopecia [99]	Placebo controlled, randomized, evaluator-blinded, half-head group study in humans (AGA)	27	Micrografts enriched with HF-MSCs	- Improvement in the mean hair count was noted after 58 weeks of 18.0 hair. - The mean increase in total hair density was 23.3 hairs per cm^2^.	Six patients exhibited dynamic hair loss after 26 months.
**Bone marrow derived stem cells (BMSC)**					
Stem cell therapy as a novel therapeutic intervention for resistant cases of alopecia areata and androgenetic alopecia [56]	Double randomized clinical human study (AGA and AA)	40 (20 AGA and 20 AA)	Autologous bone marrow derived mononuclear cells (BMMCs or autologous follicular stem cells (FSC).	- Clinically, there was a considerable improvement six months following stem cell therapy injection, which was validated by immunostaining and digital dermoscopy. - The mean improvement was “very good” across all groups. - In either form of alopecia, there was no significant difference between the two procedures.	No adverse effects were noted
Application of mesenchymal stem cells derived from bone marrow and umbilical cord in human hair multiplication [109]	Experimental study in vitro and on athymic mice	-	Culture expanded MSCs from bone marrow and umbilical cord of human beings	The DPLTs created in this technique were identical in size, shape and expression to actual DP.	MSCs were preconditioned in dermal papilla formation medium (DPFM) before being subcultured to create self-aggregated DPLTs.
**Umbilical cord blood derived cells**					
Human umbilical cord blood mesenchymal stem cells engineered to overexpress growth factors accelerate outcomes in hair growth [57]	Experimental study on C3H/HeJ mice	-	hUCB-MSCs	- hUCB-MSCs accelerated anagen initiation and hair follicle neogenesis. - Co-culture with hUCB-MSCs increased the viability of human dermal papilla cells (hDPCs) and up-regulated its hair induction-related proteins. - hUCB-MSCs stimulate hair growth through a paracrine mechanism.	IGFBP-1 had a beneficial influence on cell survival, VEGF secretion, alkaline phosphatase (ALP), CD133, and b-catenin expression, and the formation of 3D spheroids of hDPCs via colocalization of an IGF-1 and IGFBP-1.
Migration Inhibitory Factor in Conditioned Medium from Human Umbilical Cord Blood-Derived Mesenchymal Stromal Cells Stimulates Hair Growth [40]	Double-blind placebo-controlled clinical trial (AGA)	30	hUCB-MSC-CM	- When compared to CM alone, primed MSC-derived CM (P-CM) with combinations of TGF-1 and LiCl significantly enhanced the viability of DPCs. - P-CM increased hair density by 14.24 percent (*p* = 0.001). - At 16 weeks, there was a statistically significant increase in hair thickness and rate of hair growth.	- The macrophage migration inhibitory factor (MIF) in the P-CM released by MSCs influenced the secretion of vascular endothelial growth factor (VEGF) in DPCs, which acts via VEGF-related β-catenin and P-GSK-3β [SER9] signaling pathway - P-CM, through a paracrine mechanism, can boost hair growth efficacy.
**Wharton jelly stem cells**					
Human Wharton’s Jelly Mesenchymal Stem Cells Plasticity Augments Scar-Free Skin Wound Healing with Hair Growth [139]	Experimental study on black SCID mice	-	Human WJ-MSCs	- During long-term culture, human Wharton’s Jelly-derived MSCs (WJ-MSC) retained their phenotypic characteristics and in vitro differentiation plasticity. - During long-term in vitro cultures, human WJMSCs retained several inherent MSC properties. - In the presence of pro-inflammatory cytokines, perinatal MSCs exhibited higher quantities of immunomodulatory molecules than human bone marrow-derived MSC (BM-MSC). - WJ-MSCs sown on decellularized amniotic membrane improved scar-free wound healing and hair growth. - The biomechanical parameters of regenerated skin tissue from SCID mice transplanted with WJ-MSC seeded on decellularized amniotic membrane were the best.	- Human platelet lysate was successfully employed to grow isolated WJ-MSCs from human Wharton jelly tissue of the umbilical cord. - The fetal MSCs showed more stemness compared to gold standard adult bone marrow derived MSCs
Wharton’s jelly-derived mesenchymal stem cells in the treatment of four patients with alopecia areata [58]	Experimental case series on humans	4	Human WJ-MSC	- Hair regrowth was noted in all patients at the areas where the cell suspension was delivered by an average of 67%. - After the first 12 weeks, all patients had significantly more hair regrowth (I12- mean 52.2%) than in the subsequent 12 weeks (I24- mean 32%)	This intervention was determined to be risk-free, with no negative side effects.
**Amniotic fluid Mesenchymal stem cells (AF-MSC)**					
Secretory Profiles and Wound Healing Effects of Human Amniotic Fluid–Derived Mesenchymal Stem Cells [41]	Experimental study in vitro and on ICR mice	-	AF-MSCs	- Through the TGF/SMAD2 pathway, use of AF-MSC-CM dramatically accelerated wound healing by dermal fibroblasts. - AF-MSCs, like BM-MSCs, can differentiate into adipogenic, osteogenic and chondrogenic lineages. - When treated with AF-MSC-CM, the number of viable dermal fibroblasts was dramatically increased. - AF-MSCs secrete a high quantity of growth factors and cytokines, which aid in wound healing. - Quantification of migrated cells demonstrated a considerable increase in dermal fibroblasts’ migratory capacity following incubation with varied AF-MSC-CMs. - AF-MSC-CM dramatically accelerated wound closure in mice in vivo.	AF-MSC-CM could be a potential therapeutic for improving the efficacy of tissue repair.
Overexpression of Nanog in amniotic fluid–derived mesenchymal stem cells accelerates dermal papilla cell activity and promotes hair follicle regeneration [159]	Experimental study on C57BL/6 mice	-	AF-N-MSCs	- CM produced from AF-N-MSCs (AF-N-CM) accelerated the telogen-to-anagen transition and boosted HF density in hair follicles (HFs). - The expression of DP and HF stem cell markers, as well as genes associated with hair induction, was significantly higher in AF-N-CM than in AF-MSC-derived CM.	The secretome of autologous MSCs genetically modified to overexpress Nanog could be an attractive option for treatment of alopecia as a potent anagen inducer and hair growth stimulator.

## Data Availability

Not applicable.
